# p53-regulated autophagy is controlled by glycolysis and determines cell fate

**DOI:** 10.18632/oncotarget.5218

**Published:** 2015-08-19

**Authors:** Lei Duan, Ricardo E. Perez, Batzaya Davaadelger, Elena N. Dedkova, Lothar A. Blatter, Carl G. Maki

**Affiliations:** ^1^ Department of Anatomy and Cell Biology, Rush University Medical Center, Chicago IL, USA; ^2^ Department of Molecular Biophysics and Physiology, Rush University Medical Center, Chicago, IL, USA

**Keywords:** p53, Nutlin-3a, glycolysis, autophagy

## Abstract

The tumor suppressor p53 regulates downstream targets that determine cell fate. Canonical p53 functions include inducing apoptosis, growth arrest, and senescence. Non-canonical p53 functions include its ability to promote or inhibit autophagy and its ability to regulate metabolism. The extent to which autophagy and/or metabolic regulation determines cell fate by p53 is unclear. To address this, we compared cells resistant or sensitive to apoptosis by the p53 activator Nutlin-3a. In resistant cells, glycolysis was maintained upon Nutlin-3a treatment, and activated p53 promoted prosurvival autophagy. In contrast, in apoptosis sensitive cells activated p53 increased superoxide levels and inhibited glycolysis through repression of glycolytic pathway genes. Glycolysis inhibition and increased superoxide inhibited autophagy by repressing ATG genes essential for autophagic vesicle maturation. Inhibiting glycolysis increased superoxide and blocked autophagy in apoptosis-resistant cells, causing p62-dependent caspase-8 activation. Finally, treatment with 2-DG or the autophagy inhibitors chloroquine or bafilomycin A1 sensitized resistant cells to Nutlin-3a-induced apoptosis. Together, these findings reveal novel links between glycolysis and autophagy that determine apoptosis-sensitivity in response to p53. Specifically, the findings indicate 1) that glycolysis plays an essential role in autophagy by limiting superoxide levels and maintaining expression of ATG genes required for autophagic vesicle maturation, 2) that p53 can promote or inhibit autophagy depending on the status of glycolysis, and 3) that inhibiting protective autophagy can expand the breadth of cells susceptible to Nutlin-3a induced apoptosis.

## INTRODUCTION

P53 is a stress-responsive transcription factor and potent tumor suppressor. It has been known for many years that p53 can kill cancer cells or inhibit their growth by inducing genes that promote apoptosis (e.g. *PUMA, Noxa, Bax*) or cell cycle arrest (*P21*) [1-4]. However, more recent studies indicate p53 can also disrupt cancer metabolism by regulating expression of various glycolytic and other metabolic pathway genes [5-9]. The ability of p53 to disrupt cancer metabolism is considered essential for its tumor suppressor function. Cancer cells often display increased glycolysis and an increased dependence on glycolysis for ATP production. P53 can inhibit glycolysis by repressing multiple glycolysis pathway genes, while at the same time promoting a switch to oxidative phosphorylation through increased expression of factors like cytochrome oxidase 2 (SCO2) [8, 9]. Zawacka-Pankau et al. (2011) reported that the ability of p53 to inhibit glycolysis is important for p53-mediated apoptosis [10]. How glycolysis inhibition by p53 contributes to tumor cell apoptosis and/or tumor suppression remains unknown.

In addition to regulating apoptosis, cell cycle arrest, and metabolism, p53 can also regulate genes that control autophagy. Autophagy is a process of “self-eating” in which damaged organelles, misfolded proteins, and other components are broken down and degraded in lysosomes [11-13]. This degradation serves at least three purposes: first, it allows the recycling/reuse of essential cellular building blocks needed for survival; second, it can promote or maintain energy levels by shuttling autophagic breakdown products into energy-producing metabolic pathways; third, it can inhibit apoptosis through degradation of factors such as p62, which might otherwise mediate activation of apoptotic caspases, like caspase-8 [14]. Autophagy is regulated, in part, by the AMPK-mTORC1 energy and nutrient sensing pathway [15, 16]. mTORC1 normally inhibits autophagy by phosphorylating and inhibiting ULK1 and 2 [17], which are components of the autophagy-initiating complex. AMPK becomes activated in response to low nutrient or energy levels [18, 19]. Activated AMPK then phosphorylates TSC2, which inhibits mTORC1 [20], or directly phosphorylates the mTORC1 binding partner Raptor to inhibit mTOCR1 activity [21]. mTORC1 inhibition leads to activation ULK1/2 and initiation of autophagy [16]. AMPK can also directly activate ULK1/2 by phosphorylation [17]. p53 can activate AMPK and inhibit mTORC1. For example, p53 can increase expression of Sestrin-1 [22], an anti-oxidant that promotes AMPK activation, and can also activate AMPK by increasing expression of the *AMPKα/β* subunits [23]. AMPK activation by p53 leads to inhibition of mTORC1 and a subsequent increase in autophagy.

Metabolic stress caused by nutrient deprivation induces autophagy that in most circumstances promotes survival by generating nutrients [24-28]. However, the effect of glucose deprivation on autophagy is less clear. For example, Marambio et al (2010) reported glucose deprivation increased autophagy in cultured cardiac myocytes, suggesting autophagy could be a pro-survival mechanism when glucose levels are low. In contrast, Ramirez-Pinedo et al reported that autophagic flux was decreased in glucose-deprived cells, and that autophagy inhibitors did not protect cells from death caused by glucose starvation [29]. In addition, Moruno-Manchón et al found that glucose addition stimulated autophagy under serum-starvation conditions [30]. These latter findings suggested glucose metabolism (e.g. glycolysis) can promote autophagy, though the mechanism of autophagy stimulation by glucose is not clear. Notably, glucose deprivation can induce mitochondrial dysfunction and increase reactive oxygen species (ROS) [31, 32]. ROS has been reported to both inhibit and promote autophagy [31, 33, 34]. The extent to which ROS might inhibit autophagy in glucose deprived cells has not been determined. Finally, as noted above p53 can repress glycolytic genes and inhibit glycolysis. This, conceivably, could increase ROS levels and subsequently promote or inhibit autophagy.

Wild-type p53 is normally expressed at low levels and inactive due to MDM2, an E3 ligase that binds p53 and promotes its degradation. MDM2 antagonists have emerged as potential therapeutic drugs for cancers with wild-type p53 [35-37]. These compounds block MDM2 binding to p53, thus unleashing p53 to kill and/or inhibit cancer cell growth. Nutlin-3a (Nutlin) is the prototype MDM2 antagonist first described in 2004 [38]. Nutlin occupies the p53-binding site in MDM2, blocking the interaction between p53 and MDM2 and stabilizing/activating p53. Nutlin and its derivatives showed considerable promise in pre-clinical studies and recently entered clinical trials. However, resistance to MDM2 antagonists (e.g. Nutlin and derivatives) is an emerging problem that could limit their clinical effectiveness [39, 40]. For example, some p53 wild-type cancer cells undergo apoptosis as their primary response to Nutlin while others are largely resistant to apoptosis and undergo growth/cell-cycle arrest. We and others showed growth/cell-cycle arrest induced by Nutlin is reversible and in some cases can give rise to therapy-resistant tetraploid cells [41]. Targeting resistant cells to apoptosis would increase the therapeutic potential of MDM2 antagonists like Nutlin and its derivatives. The molecular basis for resistance to Nutlin-induced apoptosis has not been clarified.

We wished to determine if differences in glycolysis and/or autophagy could explain differences in cancer sensitivity to Nutlin-induced apoptosis. To this end, we identified p53 wild-type cancer cell lines susceptible or resistant to Nutlin-induced apoptosis. In resistant cells, glycolysis was maintained upon Nutlin-3a treatment, and activated p53 promoted prosurvival autophagy. In contrast, in apoptosis sensitive cells activated p53 increased superoxide levels and inhibited glycolysis through repression of glycolytic genes. Glycolysis inhibition and increased superoxide inhibited autophagy by causing repression of autophagy genes essential for autophagic vesicle maturation (*ATG3, 5, 7, 10*). Importantly, glucose deprivation or 2-deoxyglucose (2-DG) caused repression of ATG gene*s* and inhibited autophagic flux in apoptosis-resistant cells, leading to p62-dependent caspase-8 activation. Finally, 2-DG or the autophagy inhibitors bafliomycin A1 and chloroquine sensitized otherwise resistant cells to Nutlin-induced apoptosis. Together, these findings demonstrate that p53-regulated autophagy is controlled by glycolysis and determines cell fate (apoptosis sensitivity) in response to activated p53.

## RESULTS

### Sensitivity to nutlin-induced apoptosis correlates with inhibition of glycolysis

**Figure 1:**
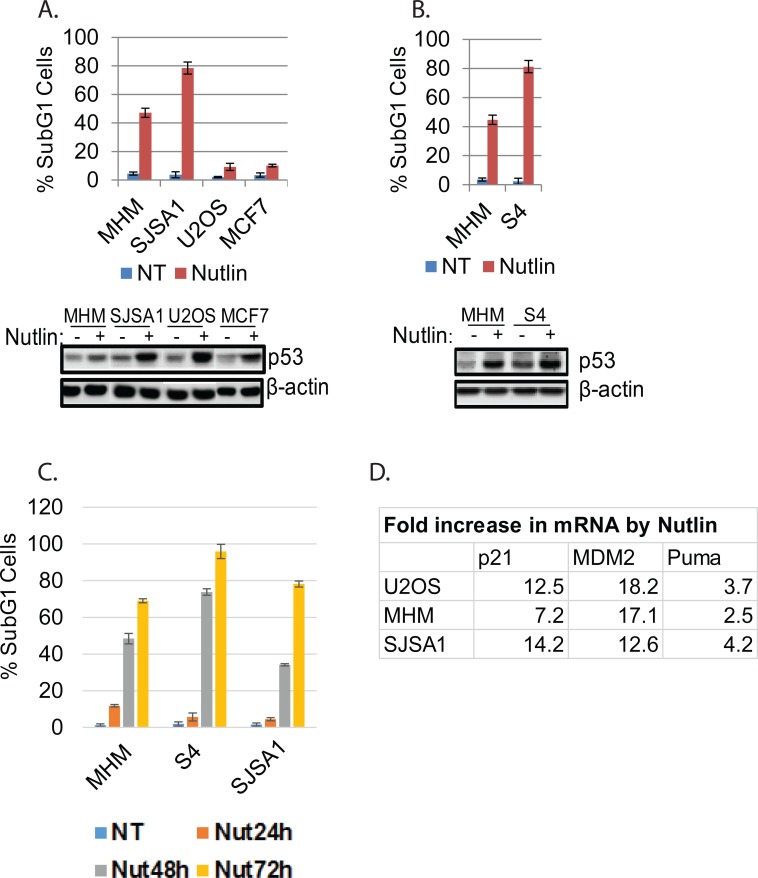
Cell lines sensitive or resistant to Nutlin-induced apoptosis **A.** and **B.** The indicated cell lines were treated with vehicle (NT) or Nutlin (10 μM) for three days and analyzed for apoptosis (Sub-G1). The average percentage of apoptotic cells from triplicate was presented as a graph with standard deviation indicated (upper). Lysates of the cells treated with vehicle or Nultin for 24 hours were immunoblotted for p53 and β-actin (lower). **C.** The indicated cell lines were treated with vehicle (NT) or Nutlin (10 μM) for a time course and analyzed for apoptosis (Sub-G1). **D.** The indicated cell lines were treated with vehicle (NT) or Nutlin (10 μM) for 24 hours and mRNA was analyzed for the indicated genes.

Small-molecule MDM2 antagonists (e.g Nutlin and derivatives) are being developed as therapeutics for cancers with wild-type p53. However, some p53 wild-type cancer cells undergo apoptosis in response to Nutlin, while others are largely resistant to apoptosis and undergo cell-cycle arrest as their primary response. The molecular basis for resistance to Nutlin-induced apoptosis is unknown. To address this, we tested cancer cell lines for their relative sensitivity to Nutlin-induced apoptosis. P53 was induced by Nutlin in cell lines expressing WT p53 (MHM, SJSA1, MCF7, U2OS, Figure 1A) but not p53-null cells or cells with mutated p53 (Supplemental Figure 1A and 1B). MHM and SJSA1 cells were sensitive to Nutlin-induced apoptosis (determined by %Sub-G1 cells, Figure 1A) whereas U2OS and MCF7 were largely resistant and underwent cell-cycle arrest in response to Nutlin. In other studies, we isolated MHM cells that survived repeated cisplatin exposure. These cells (called S4 cells) are more sensitive to Nutlin-induced apoptosis than parental MHM (Figure 1B). Nutlin did not induce significant apoptosis at 24 hours in MHM, S4, or SJSA1 cells (Figure 1C) we therefore chose the 24 hour time point for further analysis of signaling changes. Notably, Nutlin induces similar expression of p21, MDM2, and Puma in sensitive and resistant cells (Figure 1D and data not shown).

**Figure 2:**
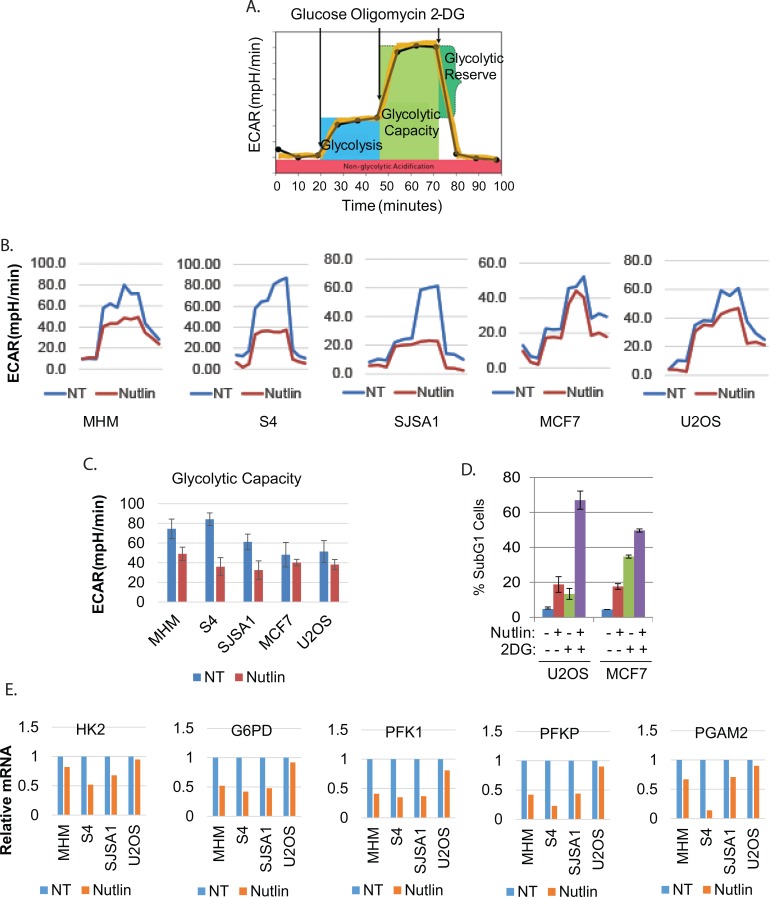
Nutlin reduces glycolysis and glycolytic genes in sensitive but not resistant cells **A.** Graphic illustration of measurement of ECAR by Seahorse machine. **B.** The indicated cell lines were treated with vehicle (NT) or Nutlin (10 μM) for 16 hours and analyzed by Seahorse Extracellular Flux Analyzer for ECAR. **C.** Average glycolytic capacity from 6 replicates was presented as a graph with standard deviated indicated. There is significant difference between vehicle treated and Nutlin-treated MHM, S4, and SJSA1 cells (*p* < 0.01) but no difference for MCF7 and U2OS cells (*p* > 0.05). D. U2OS and MCF7 cells were treated with vehicle, Nutlin, 2-DG, or Nutlin plus 2-DG for three days and analyzed for apoptotic cells (Sub-G1). The average percentage of apoptotic cells from triplicate was presented as a graph with standard deviation indicated. **D.** The indicated cell lines were treated with vehicle (NT) or Nutlin (10 μM) for 24 hours. Relative mRNA of the indicated genes was quantitatively analyzed by Real-Time PCR and presented as graphs.

Cancer cells undergo metabolic reprogramming that often includes an increased dependency on glycolysis. In previous studies, glycolysis was implicated in cell survival in response to p53 activators [10]. We therefore asked if glycolysis was regulated differently in cells resistant or sensitive to Nutlin-induced apoptosis. For this, we monitored extracellular acidification rate (ECAR) (using Seahorse Extracellular Flux Analyzer) as an indicator of glycolysis and glycolytic capacity (illustrated in Figure 2A). Nutlin caused pronounced inhibition of glycolysis and glycolytic capacity in MHM, SJSA1 and S4 cells, but had relatively little effect in Nutlin-resistant U2OS, and MCF7 cells (Figure 2B, 2C). This suggests glycolysis inhibition may be a general feature of Nutlin-sensitive cells, and maintaining glycolysis may contribute to Nutlin resistance. To examine this further, we monitored apoptosis (%sub-G1 cells) in U2OS and MCF7 cells co-treated with Nutlin and the glucose-metabolism inhibitor 2-DG (Figure 2D). 2-DG increased Nutlin-induced killing in both cell lines, suggesting glucose metabolism (e.g. glycolysis) is required for survival. Next, we examined expression of multiple glycolysis and glucose metabolism genes in MHM, SJSA1, S4, and U2OS cells untreated or treated with Nutlin. These studies revealed *HK2, G6PD, PFK1, PFKP*, and *PGAM2* as the most strikingly different between MHM, SJSA1, S4, and U2OS cells. Hexokinase-2 (HK2) converts glucose to glucose-6P, the first step in glycolysis. G6PD promotes glucose shunting to the pentose phosphate pathway (PPP). Phosphofructokinases PFK1 and PFKP convert fructose-6P to fructose 1,6-P. PGAM2 promotes conversion of 3-phosphoglycerate (3-PGA) to 2-phosphoglycerate (2-PGA) in the glycolytic pathway. *HK2, G6PD, PFK1, PFKP*, and *PGAM2* expression were repressed in Nutlin treated S4, SJSA1 and MHM cells but not U2OS cells (Figure 2E). Downregulation of these genes is specific because other glycolytic genes such as PFK2, TIGAR, and PARKIN were not downregulated by Nutlin (Supplemental Figure 1C). Knockdown of p53 prevented repression of these genes in Nutlin-treated S4 cells (Supplemental Figure 1D and 1E). In p53-null H1299 and MG63 cells Nutlin did not affect expression of G6PD, PFK1, and PFKP (Supplemental Figure 1F). The results suggest inhibition of glycolysis/glucose metabolism in MHM, SJSA1, and S4 cells results from Nutlin-induced repression of particular glycolysis pathway genes dependent on p53.

### Glycolysis regulates autophagic flux and survival in response to Nutlin

**Figure 3:**
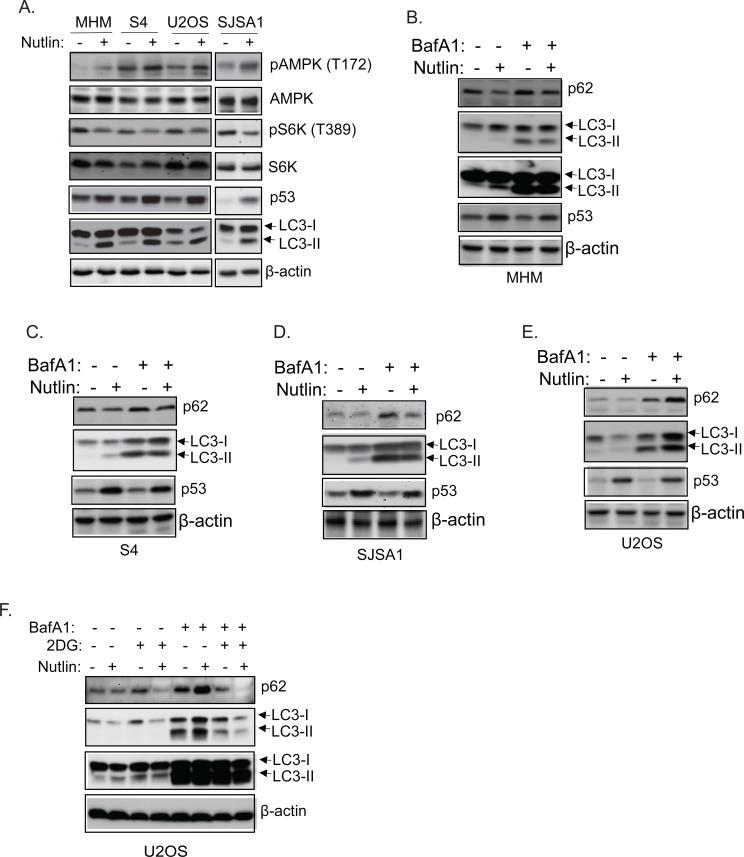
Nutlin inhibits autophagic flux in sensitive cells but promotes autophagic flux in resistant cells dependent on glycolysis **A.** The indicated cell lines were treated with vehicle or Nutlin (10 μM) for 24 hours. Whole cell lysates were immunoblotted for phospho-AMPK (T172) and total AMPK, phospho-p70S6K or p70S6K, p53, LC3, and β-actin. MHM **B.**, S4 **C.**, SJSA1 **D.**, and U2OS **E.** cells were treated with vehicle, Nutlin (10 μM), Bafilomycin A1 (BafA1, 10 μM), or Nutlin plus BafA1 for 24 hours. Whole cell lysates were immunoblotted for p62, LC3, p53, and β-actin. F. U2OS cells were treated with vehicle, Nutlin, 2-DG, or 2-DG plus Nutlin in the presence or absence of BafA1 for 24 hours. Whole cell lysates were immunoblotted for p62, LC3, and β-actin.

Autophagy increases and can promote survival in response to low energy or low nutrient conditions. Autophagy is regulated in large part by the AMPK-mTORC1 energy/nutrient sensing pathway [15, 16]. mTORC1 normally inhibits autophagy by phosphorylating and inhibiting ULK1 and ULK2, which are components of the autophagy-initiating complex. Activated AMPK inhibits mTORC1 and directly phosphorylates ULK1, thus activating ULK1/2 and stimulating autophagy. P53 can promote autophagy by activating AMPK and inhibiting mTORC1 [22]. We therefore asked if differences in autophagy were evident in cells resistant or sensitive to apoptosis by Nutlin. LC3-II and p62 are autophagy factors that are degraded in autophagic lysosomes. In MHM, S4, SJSA1, and U2OS cells Nutlin treatment increased phosphorylation of AMPK at T172 (activating phosphorylation) and decreased phosphorylation of p70S6K (indicative of decreased mTORC1 activity) (Figure 3A). This was accompanied by increased expression of LC3-II and decreased p62 (Figures 3A-3E). These results are consistent with previous findings and suggest that Nutlin-induced p53 can activate AMPK and promote autophagy. Bafilomycin A1 disrupts autophagosomes and inhibits autophagic protein degradation, including degradation of LC3-II and p62. Thus, the extent to which LC3-II and p62 increase in response to bafilomycin A1 reflects the rate with which autophagic degradation occurs, or “autophagic flux”. We found LC3-II and p62 increased in MHM, SJSA1, S4, and U2OS cells treated with bafilomycin A1 alone, indicating autophagic degradation was occurring (Figure 3B-3E). However, LC3-II and p62 levels increased to a greater extent in U2OS cells co-treated with Nutlin plus bafilomycin A1 compared to cells treated with bafilomycin A1 alone (Figure 3E). This indicates Nutlin treatment increased autophagic flux in U2OS cells. In contrast, LC3-II and p62 levels increased to a lesser extent in MHM, S4 and SJSA1 cells co-treated with Nutlin plus bafilomycin A1 vs cells treated with bafilomycin A1 alone (Figure 3B-3D) indicating that Nutlin treatment decreased autophagic flux in MHM, SJSA1 and S4 cells. Finally, to ask if glycolysis plays a role in autophagy upon Nutlin treatment, U2OS cells were treated with 2-DG, and simultaneously treated with Nutlin and bafilomycin A1. The results showed that LC3-II and p62 levels increased to a lesser extent in U2OS cells co-treated with 2-DG plus Nutlin and bafilomycin A1 vs cells treated with Nutlin plus bafilomycin A1 alone (Figure 3F). This suggests that the Nutlin-induced increase in autophagic flux in U2OS cells is dependent on glycolysis, and that glycolysis inhibition contributes to the reduction in autophagic flux in MHM, SJSA1, and S4 cells treated with Nutlin.

**Figure 4:**
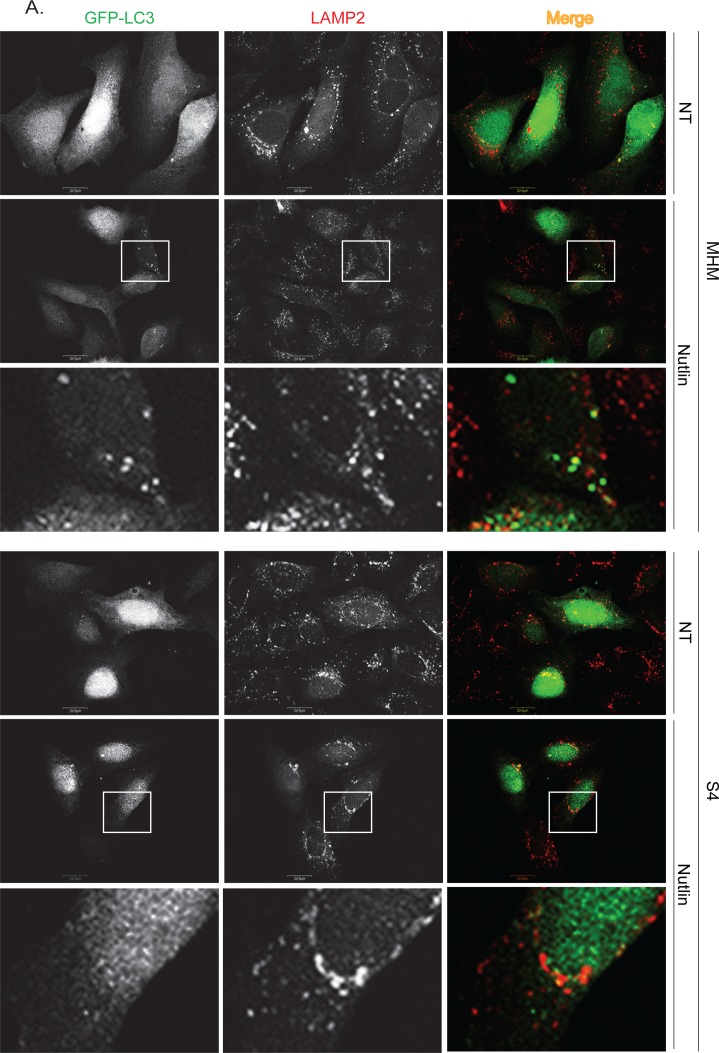

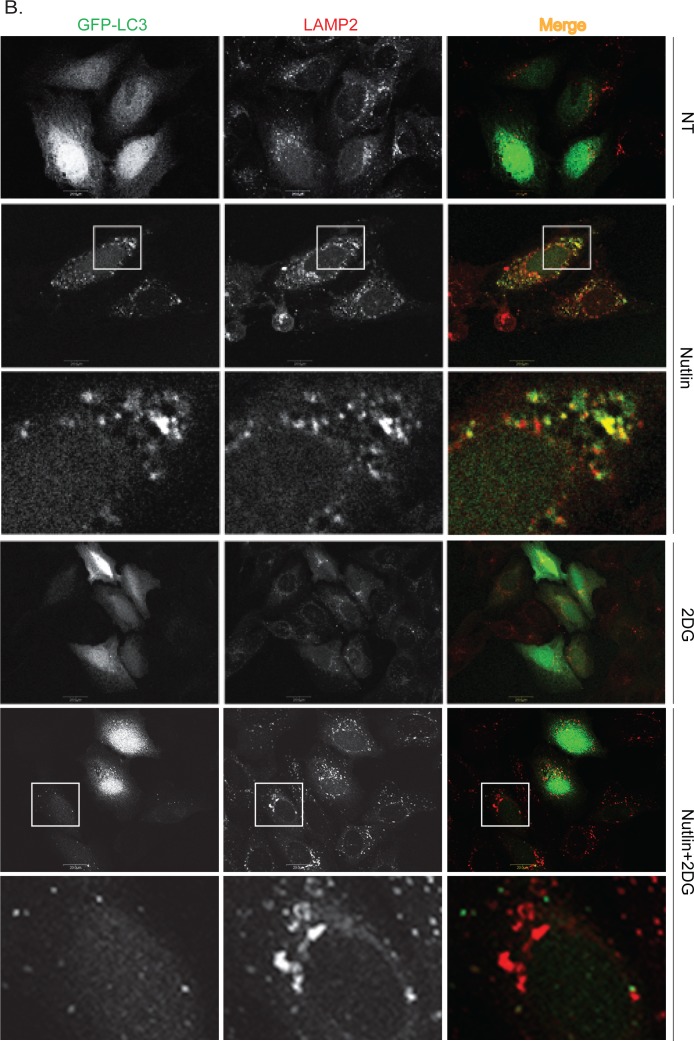
Nutlin affects formation of GFP-LC3-labeled autophagosomes differently in apoptosis sensitive and resistant cells **A.** MHM and S4 cells were transiently transfected with GFP-LC3 for 24 hours and subsequently treated with vehicle (NT) or Nutlin (10 μM) for 24 hours. The cells were fixed and immunostained for LAMP2 and then scanned with a confocal microscope. The representative images for intracellular GFP-LC3 (green) and LAMP2 (red) were shown (the size of scale bar is 20 μM). **B.** U2OS cells were transiently transfected with GFP-LC3 for 24 hours and subsequently treated with vehicle (NT) Nutlin (10 μM), 2-DG (0.1 M), or Nutlin plus 2-DG for 24 hours. The cells were fixed and immunostained for LAMP2 and then scanned with a confocal microscope. The representative images for intracellular GFP-LC3 (green) and LAMP2 (red) were shown (the size of scale bar is 20 μM).

**Figure 5:**
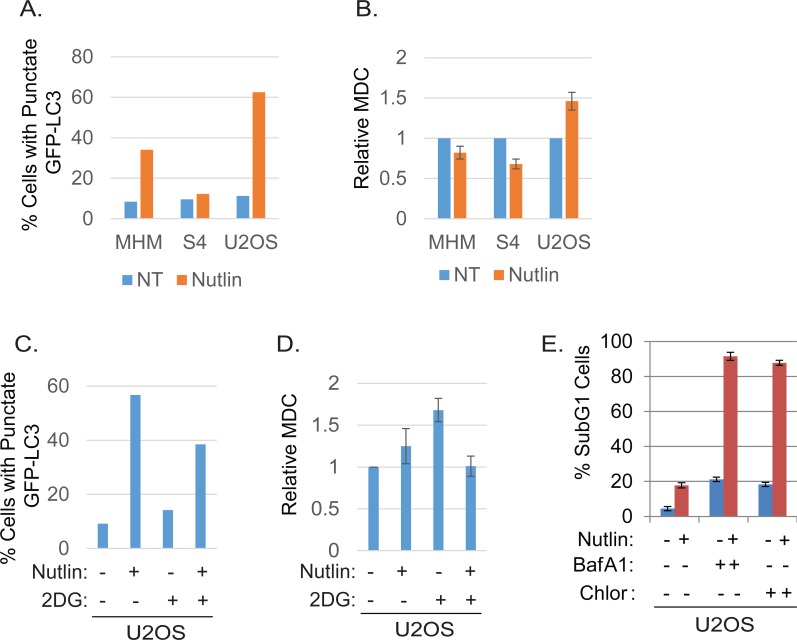
Nutlin increases autophagosomes in resistant cells dependent on glycolysis Inhibition of autophagy sensitizes resistant cells to Nutlin-induced apoptosis. **A.** MHM, S4, and U2OS cells were transiently transfected with GFP-LC3 for 24 hours and subsequently treated with vehicle (NT) or Nutlin (10 μM) for 24 hours. The cells were scanned by a confocal microscope for GFP-LC-labeled autophagosomes. The percentage of the cells that showed punctate GFP-LC3 ( > 200 GFP-LC3 positive cells) were presented as a graph (representative of three independent experiments). **B.** MHM, S4, and U2OS cells were treated with vehicle (NT) or Nutlin (10 μM) for 24 hours. The cells were labelled with MDC and equal amount of cells were analyzed for MDC fluorescence. Average relative MDC fluorescence from triplicate was presented as a graph with standard deviation indicated. **C.** U2OS cells were transiently transfected with GFP-LC3 for 24 hours and subsequently treated with vehicle (NT) or Nutlin (10 μM) for 24 hours. The cells were scanned by a confocal microscope and percentage of the cells with formation of punctate GFP-LC3 in more than 200 GFP-LC3 positive cells were presented as a graph (representative of three independent experiments). **D.** U2OS cells were treated with vehicle (NT), Nutlin (10 μM), 2-DG (0.1 M), or Nutlin plus 2-DG for 24 hours. The cells were labelled with MDC and equal amount of cells were analyzed for MDC fluorescence. Average relative MDC fluorescence from triplicate was presented as a graph with standard deviation indicated. **E.** U2OS cells were treated with vehicle, Nutlin (10 μM), BafA1 (10 μM), Chloroquine (0.1 mM), or Nutlin plus BafA1 or Chloroquine for three days. The cells were analyzed for apoptosis (Sub-G1). Average percentage of apoptotic cells from triplicate was presented as a graph with standard deviation indicated.

**Figure 6:**
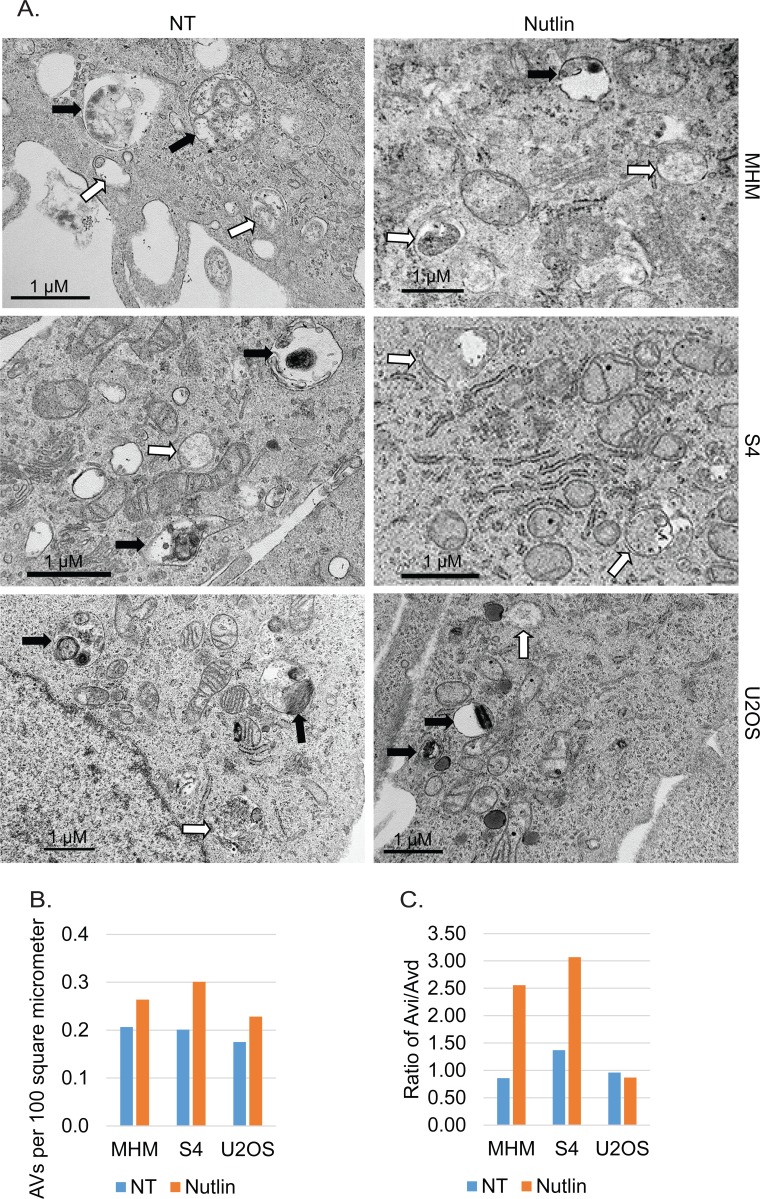
EM analysis of autophagic vacuoles **A.** MHM, S4, and X2 cells were treated with vehicle (NT) or Nutlin (10 μM) for 24 hours. The cells were analyzed by a transmission microscope to visualize autophagic vacuoles (AVs). Representative images of early autophagic vacuoles (Avi, open arrow) and late autophagic vacuoles (Avd, filled arrow) were indicated. **B.** The autophagic vacuoles were quantified and average AVs per 100 square micrometer from whole grid scan was presented as a graph. **C.** The ratio of Avi vs. Avd is presented as a graph.

To further confirm that autophagic flux is different in resistant and sensitive cells, MHM, S4, and U2OS cells were transfected with GFP-LC3 and stimulated with Nutlin for 24 hours. LAMP2 colocalization with GFP-LC3 in punctuate cytoplasmic structures is used as an indicator of autolysosomes. The cells were therefore examined for LAMP2-GFP-LC3 colocalization by confocal microscopy. The results showed that in non-treated MHM, S4, and U2OS cells GFP-LC3 was diffusely distributed (Figures 4A, 4B). In response to Nultin, a small fraction of MHM and even smaller fraction of S4 cells showed formation of punctate GFP-LC3 which was co-localized with LAMP2 (Figures 4A and 5A). In contrast, a majority of Nutlin treated U2OS cells showed formation of punctate GFP-LC3 which are larger in size than that in MHM and S4 cells and which mostly colocalize with LAMP2 (Figure 4B and 5A). However, when U2OS cells were co-treated with 2-DG and Nutlin, the size of the punctate GFP-LC3 foci was obviously smaller and there was less GFP-LC3 colocalization with LAMP2 (Figure 4B). Quantification of GFP-LC3 positive cells showed that the percentage of cells forming punctate GFP-LC3 was significantly higher in Nutlin treated U2OS cells compared with Nutlin treated MHM and S4 cells (Figure 5A). Next, we carried out electron microscopy (EM) to evaluate the relative amount and integrity of autophagic structures after Nutlin treatment. Early-stage autophagic vesicles contain organelles that are not yet degraded, whereas late-stage autophagic vesicles contain organelles in various stages of degradation that are visualized as intense, dark structures within the vesicles. Early-stage autophagic vesicles are marked by open arrowheads and late-stage autophagic vesicles are marked by filled arrowheads in Figure 6A. Nutlin treatment increased the total number of autophagic vesicles (AVs) in MHM, S4, and U2OS cells (Figure 6B). However, quantification of early and late-stage AVs showed that Nutlin increased the ratio of early autophagosomes vs. late autophagosomes in Nutlin-treated MHM and S4 cells but not in U2OS cells (Figure 6C). These results suggest Nutlin blocked autophagosomal maturation in MHM and S4 cells, potentially accounting for the inhibition of autophagic flux in these cells. We also measured MDC fluorescence, which is used as an indicator of mature autophagosomes [42, 43]. The results showed that Nutlin reduced MDC fluorescence in MHM and S4 cells but increased MDC sequestration in U2OS cells (Figure 5B). Finally, inhibition of glycolysis by 2-DG decreased MDC fluorescence in U2OS cells treated with Nutlin (Figure 5D), and 2-DG also decreased the percentage of Nutlin treated U2OS that showed GFP-LC3 colocalization with LAMP2 (Figure 5C). These results, in combination with the biochemical data in Figure 3, indicate that glycolysis regulates autophagic flux in response to Nutlin. Therefore, we wished to ask if autophagy contributes to survival in Nutlin treated cells. To this end, U2OS cells were treated with Nutlin in the presence or absence of the autophagy inhibitors Bafilomycin A1 or Chloroquine. The results showed that Bafilomycin A1 or Chloroquine sensitized U2OS cells to Nutlin-induced apoptosis (Figure 5E).

**Figure 7:**
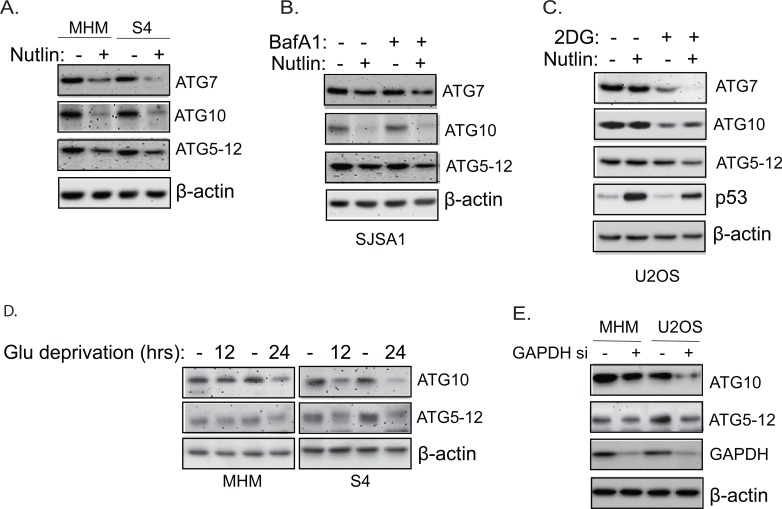
Nutlin downregulates ATG proteins in sensitive cells but not resistant cells Inhibition of glycolysis also downregulates ATG proteins. **A.** MHM and S4 cells were treated with vehicle or Nutlin (10 μM) for 24 hours. Whole cell lysates were immunoblotted for ATG7, ATG10, and ATG5-12 conjugates. Note that the anti-ATG12 antibodies detected the 50 KD ATG5-12 conjugated proteins. Similar results were also shown using a ATG5 antibody (data not shown). **B.**, SJSA1 cells were treated with vehicle or Nutlin (10 μM) in the presence or absence of BafA1 for 24 hours. Whole cell lysates were immunoblotted for ATG7, ATG10, and ATG5-12 conjugates. Note that the downregulation of ATG proteins is not blocked by BafA1 suggesting they are not degraded in lysosomes. **C.** U2OS cells were treated with vehicle or Nutlin (10 μM) in the presence or absence of 2-DG (0.1 M) for 24 hours. Whole cell lysates were immunoblotted for ATG7, ATG10, ATG5-12 conjugates, and p53. **D.** MHM and S4 cells were grow in media containing 10 mM glucose or 0.1 mM glucose (Glu deprivation) for 12 and 24 hours. Whole cell lysates were immunoblotted for ATG10 and ATG5-12 conjugates. **E.** MHM and U2OS were transfected with control siRNA or GAPDH siRNA for 48 hours. Whole cell lysates were immunoblotted for ATG10, ATG5-12 conjugates, and GAPDH with β-actin as control.

To understand how Nutlin and glycolysis regulates autophagy, we examined expression of ATG genes that regulate autophagy in the cells. The results showed that both the mRNA and protein levels of ATG5, ATG7, and ATG10 are significantly downregulated by Nutlin in apoptosis-sensitive MHM, SJSA1, and S4 cells but not in resistant U2OS cells (Figure 7A-7C and supplemental Figure 2A). The ATG5-12 conjugate, which is regulated by ATG10, was also reduced in MHM, S4, and SJSA1 cells but not U2OS cells (Figure 7A-7C). Inhibition of glycolysis in U2OS cells by 2-DG treatment decreased ATG10 mRNA and protein and to a lesser extent ATG7 protein, but did not decrease ATG5 and ATG12 mRNA (Figure 7C and Supplemental Figure 2B). Deprivation of glucose in MHM and S4 cells also reduced ATG10 mRNA and protein as well as ATG5-12 conjugated protein (Figure 7D and Supplemental Figure 2C). Notably, knockdown of GAPDH, a rate-limiting enzyme in glycolysis, also reduced ATG10 and ATG5-12 conjugate proteins (Figure 7E). The results suggest that Nutlin downregulates ATG7 and ATG10 by inhibiting glycolysis, while downregulation of ATG5 in Nutlin treated cells occurs through a mechanism independent of glycolysis inhibition. Downregulation of the glycolytic genes and ATG genes is unlikely a result of cell undergoing apoptosis because Nutlin plus Bafilomycin A1 induced massive apoptosis (Figure 5E) in U2OS cells but did not cause downregulation of ATG10 and G6PD, PFK1, and PFKP (Supplemental Figure 2D)

Notably, inhibition of glycolysis by 2DG in non-cancerous MCF10A mammary epithelial cells and three different human fibroblasts AG1522, GM8429, and GM5758 only marginally increased Nutlin-induced apoptosis compared with U2OS and MCF7 cancer cells (Supplemental Figure 5). Inhibition of autophagy by Bafilomycin A1 by itself induced significant apoptosis in GM8429 and GM5758 cells while only marginally increased Nutlin-induced apoptosis in MCF10A and AG1522 cells (Supplemental Figure 5). These results suggest that cancer cells are more sensitive to Nutlin-induced apoptosis upon inhibition of glycolysis.

### Nutlin induces aggregation of p62 and ubiquitinated proteins and activation of caspase-8 in sensitive cells and in resistant cell upon inhibition of glycolysis

**Figure 8:**
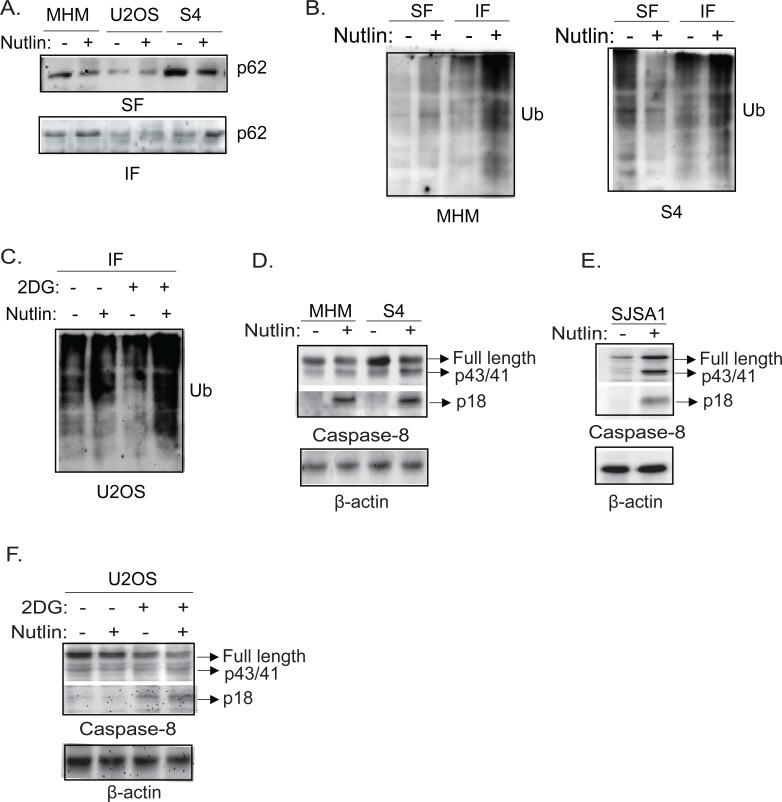
Nutlin induces accumulation of p62 and ubiquitinated proteins in insoluble fraction of lysates accompanied by activation of caspase-8 in sensitive cells 2-DG induces activation of caspase-8 in resistant cells. **A.** MHM, U2OS, and S4 cells were treated with vehicle or Nutlin for 24 hours. Equal number of cells were lysed in 1% Triton lysis buffer. The insoluble fraction (IF) and soluble fraction (SF) of the lysates were immunoblotted for p62. **B.** MHM and S4 cells were treated with vehicle or Nutlin for 24 hours. Equal amount of the SF and IF of the lysates were immunoblotted for ubiquitin. **C.** U2OS cells were treated with vehicle or Nutlin (10 μM) in the presence or absence of 2-DG (0.1 M) for 24 hours. Equal amount of the IF of the lysates were immunoblotted for ubiquitin. MHM and S4 cells **D.** and SJSA1 cells **E.** were treated with vehicle or Nutlin for 24 hours, U2OS cells **F.** were treated with vehicle or Nutlin (10 μM) in the presence or absence of 2-DG (0.1 M) for 24 hours. Whole cell lysates were immunoblotted for caspase-8 (upper part) and cleaved caspase-8 (lower part) with β-actin as loading control.

One of the functions of autophagy is to clear ubiquitinated proteins using LC3 and p62 as receptors [44]. Results from Figure 3 showed in sensitive cells that Nutlin decreased p62 levels and this reduction was not blocked by bafilomycin A1. This suggested the reduction in p62 levels in apoptosis sensitive cells does not result from increased degradation. Previous studies showed p62 can form protein aggregates that are insoluble in Triton lysis buffer and that can facilitate activation of caspase-8 [45]. We therefore asked if the distribution of p62 in soluble and insoluble fractions was altered by Nutlin, and if this was associated with activation of caspase-8. The results showed that p62 was decreased in the soluble fraction (SF) and increased in the insoluble fraction (IF) of Nutlin-treated MHM and S4 cells (Figure 8A). In contrast, p62 distribution in the soluble and insoluble fractions was unchanged by Nutlin in U2OS cells (Figure 8A). Immunoblotting of the soluble and insoluble fractions in MHM and S4 cells for ubiquitin suggested there was an increased level of ubiquitinated proteins specifically in the insoluble fraction in Nutlin treated MHM and S4 cells (Figure 8B). Moreover, in U2OS cells treated with Nutlin inhibition of glycolysis by 2-DG also led to an apparent increase in ubiquitinated proteins in the insoluble fraction (Figure 8C). These results suggest Nutlin induces aggregation of p62 and ubiquitinated proteins when glycolysis and autophagy are inhibited. P62 is known to bind and form aggregates with ubiquitinated caspase-8 that facilitate caspase-8 activation [45]. We therefore examined caspase-8 activation in the cells. Nutlin caused a pronounced increase in the level of activated caspase-8 (p18) in MHM, and S4 cells (Figure 8D) but little or no activation of caspase-8 in U2OS cells (Figure 8E). However, glycolysis inhibition by 2-DG activated caspase-8 in U2OS cells in the presence or absence of Nutlin (Figure 8E).

We further examined cellular p62 localization using GFP-labeled p62. MHM, S4, and U2OS cells were transfected with GFP-p62 and treated with Nutlin for 24 hours. The cells were fixed and immunostained for LAMP2 and then analyzed by confocal microscopy. The results show that in non-treated cells GFP-p62 was weakly visualized in cytoplasm (Supplemental Figure 3). In Nutlin treated cells the GFP-p62 fluorescence was obviously increased compared to the non-treated cells. In Nultin-treated MHM and S4 cells, GFP-p62 appears to be in patches without showing punctate structures and do not colocalize with LAMP2 (Supplemental Figure 3). However, in Nultin-treated U2OS cells, p62 formed punctate structures that colocalized with LAMP2 in a significant fraction of the cells (Supplemental Figure 3). These results suggest that p62 goes to LAMP2-positive lysosomes in resistant cells but not in sensitive cells in which they appear to form protein aggregates.

### Superoxide plays a role in the downregulation of ATG genes and suppression of autophagic flux

**Figure 9:**
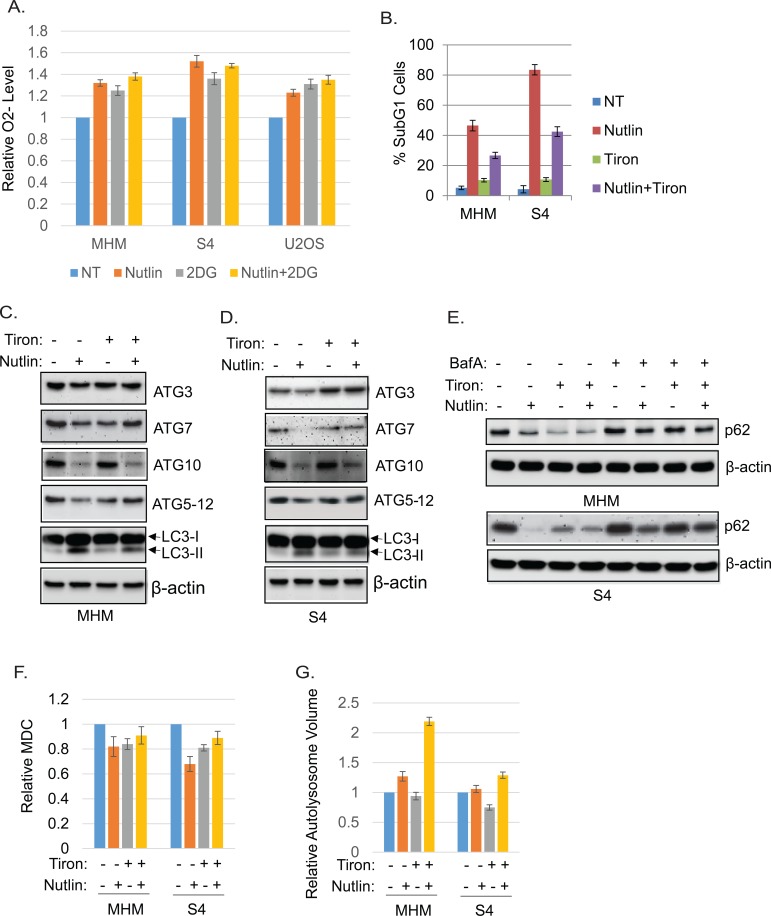
Inhibition of glycolysis induces O2- The O2- scavenger Tiron promotes ATG protein expression, autophagy, and cell survival. **A.** MHM, S4, U2OS cells were treated with vehicle, Nutlin (10 μM), 2-DG (0.1 M), or Nutlin plus 2-DG for 24 hours. Intracellular O2- was measured with DHE and average relative O2- level from triplicate was presented as a graph with standard deviation indicated. **B.** MHM and S4 cells were treated with vehicle, Nutlin (10 μM), Tiron (5 mM), or Nutlin plus Tiron for 72 hours. The cells were analyzed for apoptosis (sub-G1). Average percentage of apoptotic cells from triplicate was presented with standard deviation indicated. MHM cells **C.** and S4 cells **D.** were treated with vehicle, Nutlin (10 μM), TIron (5 mM), or Nutlin plus Tiron for 24 hours. Whole cell lysates were immunoblotted for ATG3, 7, ATG10, ATG5-12 conjugates, LC3, and β-actin. **E.** MHM cells and S4 cells were treated with vehicle, Nutlin (10 μM), TIron (5 mM), or Nutlin plus Tiron in the presence or absence of BafA1 for 24 hours. Whole cell lysates were immunoblotted for p62 and β-actin. MHM and S4 cells were treated with vehicle, Nutlin (10 μM), Tiron (5 mM), or Nutlin plus Tiron for 24 hours, the cells were analyzed for MDC sequestration and autolysosomal volume. Average relative MDC fluorescence **F.** and autolysosomal volume **G.** from triplicate were presented as graphs with standard deviation indicated.

Glycolysis is known to regulate reactive oxygen species (ROS). Glucose deprivation and 2-DG have been shown to increase intracellular superoxide (O2-) [31, 32]. We therefore asked if O2- levels were altered in Nutlin treated cells and if O2- levels affected autophagy or apoptosis sensitivity. First, we measured O2- levels in MHM, S4, and U2OS cells with or without Nutlin treatment and/or 2-DG. The results showed that O2- was increased in all of the cells in response to Nultin (Figure 9A). 2-DG alone also increased O2- and combination of Nutlin and 2-DG further increased O2- in the cells (Figure 9A). Next we tested if neutralizing O2- with the specific O2- scavenger Tiron [46-48] affects cell response to Nutlin. Tiron significantly reduced Nutlin-induced apoptosis in MHM and S4 cells (Figure 9B), suggesting the induction of O2- by Nutlin contributes to apoptosis. Next we analyzed expression of ATG mRNA and protein. We found that Tiron upregulates basal ATG3 and ATG12 mRNA and maintained ATG3, ATG7, and ATG5-12 conjugate protein levels but not ATG10 in Nutlin-treated MHM and S4 cells (Figure 9C and 9D and supplemental Figure 4). Moreover, Tiron decreased p62 levels basally and in Nutlin treated cells, and this effect was reversed by bafilomycin A1 (Figure 9E), indicating Tiron increases autophagic flux. Tiron also increased MDC fluorescence and lysosomal volume (Figure 9F and 9G). In total, these results suggest that inhibition of glycolysis can increase O2- that downregulates ATG3/12 gene expression, contributing to inhibition of autophagic flux and autophagosome/autolysosome function in apoptosis-sensitive cells.

## DISCUSSION

P53 is a stress responsive transcription factor and potent tumor suppressor. It has been known for many years that p53 can kill cancer cells or inhibit their proliferation by activating genes whose products induce apoptosis (e.g. *PUMA, Noxa, Bax*) or cell-cycle arrest (*P21*) [1-4]. Recently p53 has also emerged as a critical regulator of cell metabolism and autophagy, two intertwined processes critical for cell proliferation and survival. P53 can inhibit glycolysis and promote mitochondrial metabolism by regulating transcription of genes involved in glycolysis, mitochondrial respiration, and glutaminolysis [5-9]. The effect of p53 on autophagy is less clear. For example, p53 activated by stress can stimulate autophagy through transcriptional upregulation of factors such as TSC1 and DRAM [49, 50], and by increasing expression of SESN1 which activates AMPK and thus inhibits mTORC1 [22]. However, p53 has also been reported to inhibit autophagy under non-stressed conditions by preventing ER stress and maintaining homeostasis through a transcription-independent mechanism [51]. Because cell metabolism and autophagy are tightly coupled and p53 can affect both, it is critical to understand how p53 regulates these two processes to determine cell survival and death. In the current study we used Nutlin to activate p53 in a number of cancer cells that are resistant or sensitive to Nutlin-induced apoptosis. Our results demonstrate that p53 can either inhibit or promote autophagy dependent on its ability to inhibit glycolysis. Further, our results reveal a critical role for glycolysis and superoxide scavenging in promoting completion of prosurvival autophagy in response to p53 activation.

Nutlin treatment inhibited glycolysis in cells sensitive to Nutlin-induced apoptosis (MHM, S4, and SJSA1 cells) but not cells resistant to apoptosis (U2OS, MCF7). However, inhibiting glycolysis by 2-DG treatment sensitized the otherwise resistant U2OS and MCF7 cells to Nutlin-induced apoptosis. These findings indicate glycolysis inhibition contributes to Nutlin-induced apoptosis, and are consistent with others findings that glycolysis promotes survival in response to p53 activation. Inhibition of glycolysis in Nutlin-treated MHM, S4, and SJSA1 cells coincided with repression of multiple glycolysis pathway genes. Galina Selinanova and colleagues reported that SP1 recruits p53 to the promoter regions of glycolysis pathway genes to mediate their repression [10], including some of the glycolysis pathway genes we found were repressed in Nutlin-sensitive MHM, S4 and SJSA1 cells. The most likely scenario therefore is that p53 is recruited to and mediates repression of glycolysis pathway genes through its association with SP1, leading to inhibition of glycolysis and apoptosis in Nutlin-sensitive but not Nutlin-resistant cells.

Our results indicate glycolysis inhibition in Nutlin-treated cells increases superoxide levels. Specifically, Nutlin treatment increased superoxide levels in apoptosis-sensitive MHM and S4 cells, but to a lesser extent in apoptosis-resistant U2OS cells. However, inhibiting glycolysis by either glucose deprivation or 2-DG treatment increased superoxide levels in U2OS. The major source of superoxide is the mitochondrial electron transport chain. Loss of mitochondrial integrity can increase superoxide levels, and previous studies showed glucose deprivation can disrupt mitochondria and increase oxidative stress [31, 32]. Thus, one possibility is that glycolysis inhibition in Nutlin-treated cells causes disruption of mitochondria and a subsequent increase in superoxide levels. A second possibility concerns the levels of glycolysis-derived antioxidants. Glucose metabolized in the pentose phosphate pathway (PPP) generates NADPH, which can counteract/neutralize ROS [52]. In addition, specific glycolysis pathway metabolites (e.g. pyruvate) have reported anti-oxidant activity [53]. Thus, a second possibility is that glycolysis inhibition increased superoxide levels by reducing NADPH production by the PPP and/or reducing the levels of anti-oxidant metabolites like pyruvate.

Our data indicate that glycolysis inhibition and superoxide levels control autophagic flux in Nutlin-treated cells to determine cell fate. Autophagy is regulated by the AMPK-mTORC1 energy and nutrient-sensing pathway. mTORC1 normally inhibits autophagy by phosphorylating and inhibiting ULK1 and ULK2 [17], which are components of the autophagy-initiating complex. Activated AMPK inhibits mTORC1, thus activating ULK1/2 and stimulating autophagy initiation. Nutlin treatment induced p53, activated AMPK, and inhibited mTORC1 in MHM, S4, and U2OS cells. This is consistent with reports that p53 can promote autophagy initiation. However, we found that glycolysis inhibition and increased superoxide levels inhibited the completion of autophagy in MHM and S4 cells. Bafilomycin A1 disrupts autophagosome-autolysosome fusion and inhibits autophagic protein degradation [54, 55], including degradation of LC3-II and p62. Thus, the extent to which LC3-II and p62 increase in response to bafilomycin A1 reflects the rate with which autophagic degradation is occurring, or “autophagic flux” [55, 56]. We found LC3-II and p62 increased in MHM, S4, and U2OS cells treated with bafilomycin A1 alone, indicating autophagic degradation was occurring. However, LC3-II and p62 levels increased to a greater extent in U2OS cells co-treated with Nutlin plus bafilomycin A1 compared to cells treated with bafilomycin A1 alone. This indicates Nutlin treatment increased autophagic flux in these cells. In contrast, LC3-II and p62 levels increased to a lesser extent in MHM and S4 cells co-treated with Nutlin plus bafilomycin A1 vs cells treated with bafilomycin A1 alone indicating that Nutlin treatment diminished autophagic flux in MHM and S4 cells. Importantly, glucose deprivation or 2-DG treatment increased superoxide levels and inhibited/diminished autophagic flux in Nutlin treated U2OS cells, and the powerful superoxide scavenger Tiron partially restored autophagic flux in Nutlin-treated MHM and S4 cells. These results indicate glycolysis inhibition and increased superoxide levels are responsible, at least in part, for the inhibition of autophagy in Nutlin-treated cells. Finally, autophagy inhibitors bafilomycin A1 and chloroquine sensitized otherwise resistant U2OS cells to Nutlin-induced apoptosis. The results indicate completion of autophagy is required for cell survival in response to Nutlin. It is noteworthy that inhibition of mTOR by p53 in response to Nutlin suppresses senescence [57, 58], which coincidentally correlates with promotion of autophagy. It will be interesting to test in non-apoptotic cells if glycolysis and autophagy play a role in p53-mediated senescence and quiescence in future studies.

How does glycolysis inhibition and/or increased superoxide levels inhibit autophagy in Nutlin treated cells? We carried out electron microscopy to evaluate the relative amount and integrity of autophagic structures after Nutlin treatment. These studies confirmed that Nutlin increases both early and late autophagolysosomes in U2OS cells, but increased early autophagosomes and decreased late autophagosomesj in MHM and S4 cells. Monodansylcadaverine (MDC) is a specific fluorescent marker of mature autophagosomes [42, 43]. Nutlin increased MDC fluorescence in U2OS cells, but decreased MDC fluorescence in MHM and S4 cells. Importantly, the decreased MDC fluorescence in Nutlin-treated MHM and S4 cells was partially reversed by the superoxide scavenger Tiron. This supports the idea that superoxide induced by Nutlin may inhibit autophagy by blocking formation of intact autophagolysosomes, consistent with previous reports. LAMP2 colocalization with LC3-II and p62 in punctuate cytoplasmic structures can be used as indicator of autophagolysosomes. Nutlin increased punctuate LAMP2 colocalization with LC3-II and p62 in U2OS cells but not MHM and S4 cells, and TIRON partially restored LAMP2 colocalization with LC3-II and p62 in MHM and S4 cells. This also supports the notion that increased superoxide inhibits autophagy by blocking autophagolysosome formation in Nutlin treated cells. Next, we examined expression of ATG3, 5, 7, 10, and 12 in Nutlin-treated cells. These ATG proteins have been shown to regulate formation of autophagosomes and maturation of autolysosomes. We found *ATG5, 7*, and *10* genes were repressed by Nutlin in MHM and S4 cells, but not U2OS. Glucose deprivation or 2-DG treatment also repressed ATG10 in MHM, S4, and U2OS cells. TIRON rescued *ATG3, 5, 7,* and*12* but not *ATG10* expression in Nutlin treated MHM and S4 cells, indicating that superoxide mediates repression of *ATG3, 5, 7,* and *ATG12*. These results suggest glycolysis inhibition and superoxide inhibit autophagy by repressing expression of *ATG3, 5, 7*, *10*, and *12*. We hypothesize one or more glycolysis or redox-regulated transcription factors may mediate repression of these ATG genes in Nutlin-treated cells.

The most striking results from our experiments are that ATG10 and ATG5-12 conjugate proteins are downregulated by Nutlin in sensitive cells and by glucose deprivation or inhibition of glycolysis (knockdown of GAPDH or 2-DG) in both sensitive and resistant cells. ATG10 is the conjugating enzyme for formation of ATG5-12 conjugated proteins [59]. Recently Chen et al showed the ATG5-12 conjugate regulates autophagosome-autolysosome fusion in mammalian cells [60]. In the EM analysis we found that there were increased early stage autophagic vacuoles but decreased late stage autopagic vacuoles in Nutlin-treated MHM and S4 cells indicating the initiation of autophagy was increased but the maturation steps are inhibited. Based on our results and those of Chen [59] we propose that glycolysis promotes autophagosome-autolysosome maturation by maintaining expression of ATG genes required for autophagic vesicle maturation. We further propose that p53 can promote or inhibit autophagy depending on the status of glycolysis. If p53 inhibits glycolysis, this will lead to repression of ATG genes, a block in autophagosome-autolysosome maturation, and increased apoptosis. In contrast, if glycolysis is not inhibited then p53 will facilitate autophagy through activation of AMPK and inhibition of mTORC1.

A final question is how inhibition of autophagy increases apoptosis in Nutlin-treated cells. It is believed that p62 can promote autophagic degradation of ubiquitinated proteins such as caspase-8 by tethering the ubiquitinated proteins to LC3-II [61]. In the absence of autophagic degradation p62 can promote caspase-8 activity by recruiting ubiquitinated caspase-8 to insoluble aggresomes [45, 61]. We found p62 was localized in insoluble aggresomes with activated caspase-8 in Nutlin-treated MHM and S4 cells, but not Nutlin-treated U2OS. However, p62 was localized in insoluble aggresomes with activated caspase-8 in Nutlin-treated U2OS when autophagy was inhibited by bafilomycin A1. Thus, autophagy inhibition could increase Nutlin-induced apoptosis, in part, through p62-mediated activation of caspase-8.

**Figure 10:**
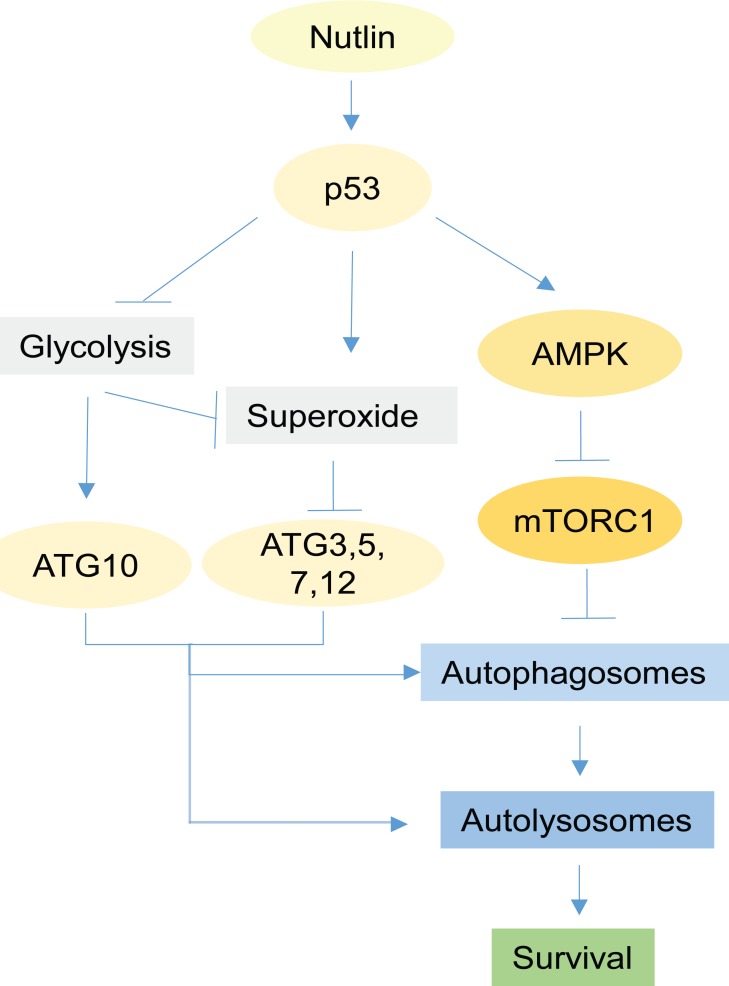
Proposed model Activation of p53 by Nutlin leads to activation of AMPK and inhibition of mTORC1 which consequently initiate autophagy by activation of ULK1/2. Autophagy is a process consisting formation of autophagosomes and fusion with lysosomes to form autolysosomes. The early steps of formation of autophagosomes is regulated by ATG7 and ATG3, E1 and E2-like enzymes respectively that conjugate phosphatidylethanolamine to LC3 in order for attachment of LC3 to membrane. Maturation of autophagosomes to autolysosomes is regulated by conjugated ATG12-ATG5 proteins for which ATG7 and ATG10 are the respective E1 and E2 enzymes. Expression of ATG10 is dependent on glycolysis while expression of ATG3, 5, 7, 12 genes is suppressed by superoxide. In Nutlin-sensitive cells p53 inhibits glycolysis and induces superoxide which leads to downregulation of ATG3, 5, 7, 10, 12 genes and consequent disruption of autophagic process demonstrated as a decrease in autophagic flux. In contrast, in Nutlin-resistant cells p53 activates autophagy without affecting glycolysis and superoxide, leading to increased autophagic flux and completion of the autophagic process. Complete autophagy promotes survival by recycling essential nutrients and inhibition of caspase-8 activation.

In total, data in this study support the model shown in Figure 10. According to this model, p53 induced by Nutlin inhibits glycolysis by repressing glycolytic pathway genes, leading to increased superoxide levels. Glycolysis inhibition and increased superoxide repress ATG3, 5, 7, 10, and 12 expression. Reduced expression of these factors inhibits formation/maturation of autophagosomes and autophagolysosomes, thus inhibiting autophagy and increasing Nutlin-induced killing. Our findings suggest a role for metabolic regulation in p53-mediated tumor suppression and apoptosis. It is becoming increasingly clear that the ability of p53 to disrupt metabolism is important for its tumor suppressor function. However, how disrupting metabolism contributes to cancer cell killing/apoptosis has not been clarified. Our findings indicate that glycolysis inhibition by p53 increases apoptosis by blocking the completion of pro-survival autophagy. Further, the findings indicate glycolysis-regulated autophagy can promote resistance to MDM2 antagonists (e.g. Nutlin), and that targeting protective autophagy could overcome this resistance and thus expand the breadth of cells susceptible to Nutlin-induced apoptosis.

## MATERIALS AND METHODS

### Cells and reagents

SJSA1, MHM, U2OS, MG63, SAOS, and MCF7 cells were obtained from ATCC. SJSA1 and MHM cells were grown in RPMI medium, MCF7, U2OS and MG63 in DMEM medium, and SAOS cells in McCoy's 5A medium with 10% fetal bovine serum (FBS), penicillin (100 U/mL) and streptomycin (100 μg/mL). Cells were plated 48h before being treated with Cisplatin (Bedford Laboratory) at the indicated concentrations. Nutlin, 2-D-glucose (2-DG), acridine orange (AO), monodansylcadaverine (MDC), bafilomycin A1, and chloroquine were obtained from Sigma Chemical Co (St. Louis, MO). MK2206 and LY2603618 were obtained from Selleck Chemicals. Oligomycin, glucose and 2-DG were prepared following manufacturer's instructions that were supplied in the XF glycolysis test kit (Seahorse Bioscience, Billerica, MA). GFP-LC3 plasmid was previously described [47]. pMXs-puro GFP-p62 (Plasmid #38277) was obtained from Addgene. Dihydroethidium (DHE) were purchased from Invitrogen.

### Immunoblotting

Whole cell extracts were prepared by scraping cells in lysis buffer (150 mM NaCl, 5 mM EDTA, 0.5% NP40, 50 mM Tris, pH 7.5), resolved by sodium dodecyl sulfate polyacrylamide gel electrophoresis (SDS-PAGE) and transferred to polyvinylidene difluoride membranes (Thermo Fisher Scientific). Antibodies to p-AMPK (T172), pan AMPK, p-S6K (T389), S6K, LC3, ATG3, ATG5, ATG7, ATG12 were from Cell Signaling; ATG10 was from MBL; p62, β-actin, and p53 (DO-1) were from Santa Cruz. Primary antibodies were detected with goat anti-mouse or goat anti-rabbit secondary antibodies conjugated to horseradish peroxidase (Life Technologies), using Clarity chemiluminescence (BIO-RAD).

### Flow cytometry

For cell cycle analysis, cells were harvested and fixed in 25% ethanol overnight. The cells were then stained with propidium iodide (25 μg/ml, Calbiochem). Flow cytometry analysis was performed on a Gallios™ Flow Cytometer (Beckman Coulter), analyzed with FlowJo 10 (Treestar Inc). For each sample, 10,000 events were collected.

### siRNA-mediated transient knockdown

GAPDH siRNA (On-target plus smart pool) and Control siRNA (On-target plus siControl non-targeting pool) were purchased from Dharmacon and were transfected according to the manufacturer's guidelines using DharmaFECT I reagent.

### shRNA-mediated stable knockdown

The lentiviral pLVUT-KRAB p53 shRNA was described (a generous gift from Dr. Patrick Aebischer) [62]; the lentiviral packaging and envelop vectors psPAX2 and pMD2G (Addgene plasmid 12260 and 12259 deposited by Dr. Didier Trono) and the pLKO-control shRNA [63] (Addgene plasmid 1864 deposited by Dr. David M. Sabatini) were obtained from Addgene plasmid repository. Lentiviral supernatants for the expression of shRNAs were generated from 293FT cells using psPAX2 and pMD2G packaging and envelope vectors according to the OpenBiosystems protocol. S4 cells were infected to establish polyclonal lines.

### RNA isolation and real-time quantitative PCR analysis

Total RNA was prepared using Total RNA Mini Kit (IBI Scientific, IA); the first cDNA strand was synthesized using High Capacity cDNA Reverse Transcription Kit (Applied Biosystems, CA). Manufacturers’ protocols were followed in each case. The PCR primers for ATG3, 5, 7, 10, 12, HK2, G6PD, PFK1, PFKP, PDGM2, and β-actin are listed in Supplemental Table-1. SYBR green PCR kit (Applied Biosystems) was used according to the manufacturer's instructions. AB7300 system was used as follows: activation at 95°C; 2 minutes, 40 cycles of denaturation at 95°C; 15 seconds and annealing/extension at 60°C; 60 seconds, followed by melt analysis ramping from 60°C to 95°C. Relative gene expression was determined by the ΔΔC_t_ method using β-Actin to normalize.

### Assay for glycolysis, glycolytic capacity and glycolytic reserve

Cells were seeded using culture media at 20,000 cells/well of XF96 cell plate (Seahorse Bioscience, Billerica, MA) 48 hours before the assay. On the day of the assay, the media was changed to DMEM (without serum, glucose or bicarbonate, but with 2 mM Glutamine), and incubated for 2 hour before the assay in a non-CO_2_ incubator at 37°C. Injections of glucose (10 mM final), oligomycin (5 μM final) and 2-DG (0.1 M final) were diluted in the DMEM media and loaded onto ports A, B and C respectively. The machine was calibrated and the assay was performed using glycolytic stress test assay protocol as suggested by the manufacturer (Seahorse Bioscience, Billerica, MA). The assay was run in one plate with 6-12 replicates. The assay was repeated at least 3 times. The rate of glycolysis is reported as extracellular acidification rate, or ECAR (mpH/min), after the addition of glucose. Glycolytic capacity is the rate of increase in ECAR after the injection of oligomycin following glucose. Oligomycin inhibits mitochondrial ATP production and therefore shifts the energy production to glycolysis with increase in ECAR revealing maximum glycolytic capacity of the cells. The glycolytic reserve is the difference between glycolytic capacity and glycolysis rate.

### Confocal immunofluorescence microscopy

For immunofluorescence analysis, cells were cultured on glass coverslips, fixed in 4% formaldehyde/PBS, permeabilized with 0.5% Triton X-100 for 5 min, and stained with anti-LAMP2 antibody followed by Alexa Fluor 564-conjugated secondary Abs. The stained cells were mounted in mounting medium (Life Technologies), and images were acquired with a confocal microscope (Olympus FluoView^TM^ 4.3) under X200 or X400 magnifications.

### Transmission electron microscopy

Preparation of pelleted sample: 2×10^6^ cells on culture dishes were rinsed with 0.1M Sorensen's Phosphate Buffer pH 7.4 three times and then fixed using 2.5% glutaraldehyde in 0.1M Sorensen's Phosphate Buffer pH 7.4 for 30 minutes. The cells were then scraped off and transferred to a 1.5 ml Eppendorf tubes and spun down in a microcentrofuge at maximal speed for 10 minutes. The cells were kept at 4°C continuously in fixation solution for 48 hours. The cells were then placed in post-fixative 1% Osmium Tetroxide and dehydrated in a series of ascending ethanol. The cells was then embedded and cured in Epoxy Resin Lx112.

Ultra microtomy with Leica Ultra-cut UCT: Thin sections of the embedded cells were cut 80nm placed on 200 mesh copper standard grids and stained with uranyl acetate and Reynolds lead citrate. The cells were then scanned on a Jeol Jem 1220 Transmission electron microscope. Digital images were acquired with Gatan Erlangshen ES 10000 W Model 785 digital camera with digital micrograph software program 1.7.1 Digital Micrograph DM.

Quantification of autophagosomes: The whole grid square was systematically scanned under the microscope for the presence of early and late autophagic vacuoles. The number of vacuoles per cell area was calculated by dividing the number of vacuoles in the grid square with the cell area in the same grid square.

### Quantitative analysis of autophagosomes and autolysosomes

Analysis of autolysosomal volume was described [47]. Briefly, cells in a 96-well plate were labeled with AO (2 μg/ml in PBS) for 10 minutes, rinsed three times with PBS, re-fed with phenol-red free media, and then analyzed for ﬂuorescence intensity using a BioTekMx microplate reader (excitation/emission of 475/520 nm for green ﬂuorescence, 475/620 nm for red ﬂuorescence). For analysis of antophagosomes/autolysosomes, MDC sequestration was conducted as previously described [47].

### Quantitative measurement of intracellular O2-

Cells were loaded with DHE (2 μM in PBS) for 30 minutes and then trypsinized. Collected cells were washed with PBS for three times, equal amount of cells were transferred to a 96-well plate, and analyzed with a BioTekMx microplate reader (excitation/emission of 510/595 nm).

### Statistical analysis

One-way analysis of variance (ANOVA) and Student's *t*-test were used to determine the statistical significance of differences among experimental groups. Student's *t*-test was used to determine the statistical significance between control and experimental groups.

## SUPPLEMENTARY MATERIAL FIGURES AND TABLE


