# Supplementary material for: p53-regulated autophagy is controlled by glycolysis and determines cell fate
Source: Oncotarget. 2015 Aug 19;6(27):23135–56. doi: 10.18632/oncotarget.5218 (PMC4695109; doi:10.18632/oncotarget.5218)
Supplement: Supplementary file 1 [file oncotarget-06-23135-s001.pdf]

p53-regulated autophagy is controlled by glycolysis and determines cell fate

Supplementary Material

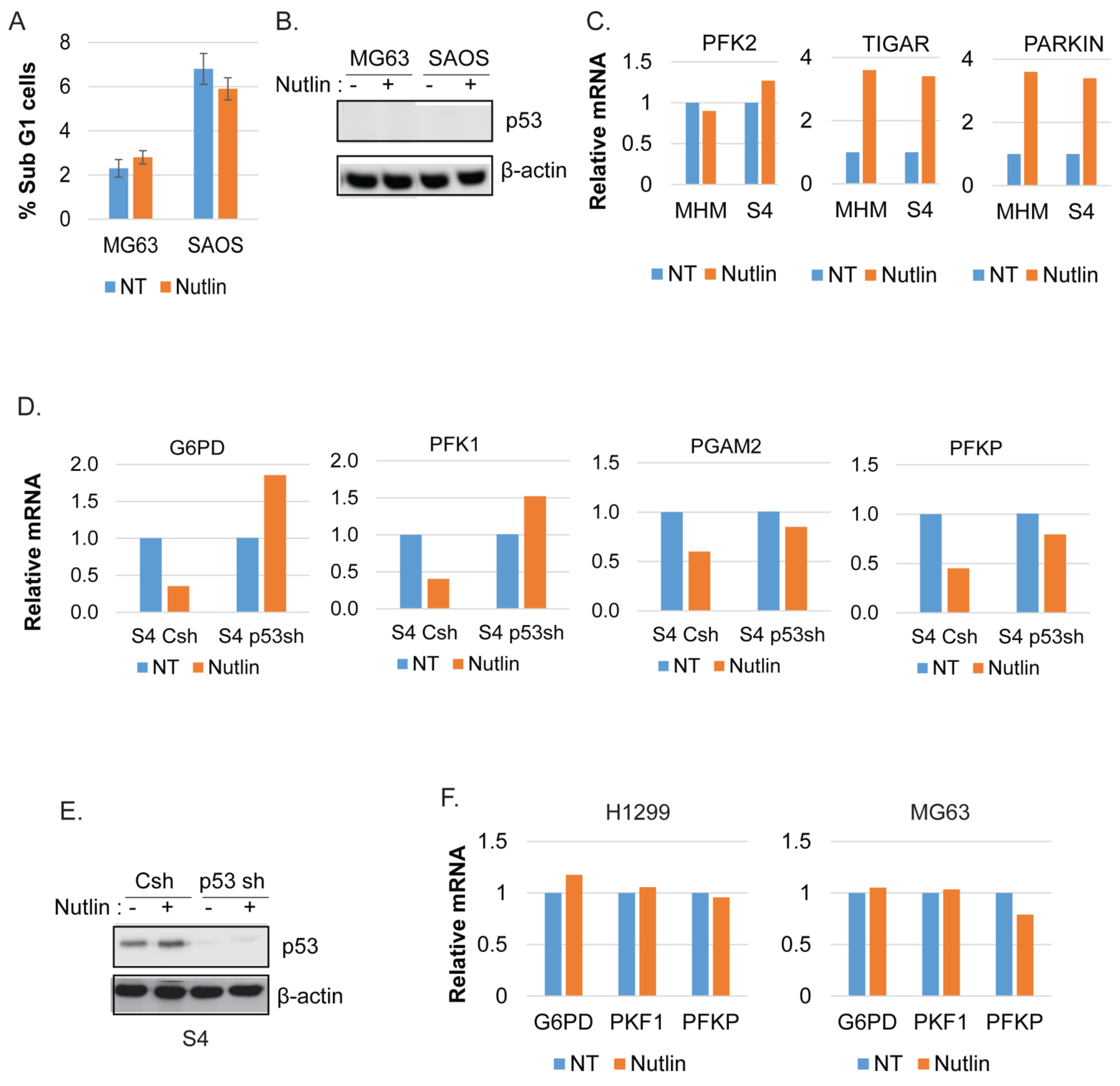

Supplemental Fig. 1. Nutlin-induced apoptosis and downregulation of glycolytic genes is dependent on p53. A. p53-null MG63 and SAOS cells were treated with vehicle (NT) or Nutlin (10 µM) for 72 hours and analyzed for sub-G1 cells. B. The cells were treated with vehicle (NT) or Nutlin for 24 hours and lysates were immunoblotted for p53 and β-actin. C. MHM and S4 cells were treated with vehicle or Nutlin for 24 hours and mRNA was analyzed for the indicated genes. D. S4 cells with stable expression of control shRNA or p53 shRNA were treated with vehicle (NT) or Nutlin for 24 hours and mRNA was quantitatively analyzed for the indicated gene expression. E. Whole cell lysates were immunoblotted for p53 and β-actin. F. p53 null H1299 and MG63 treated with vehicle or Nutlin for 24 hours and mRNA was analyzed for the indicated genes.

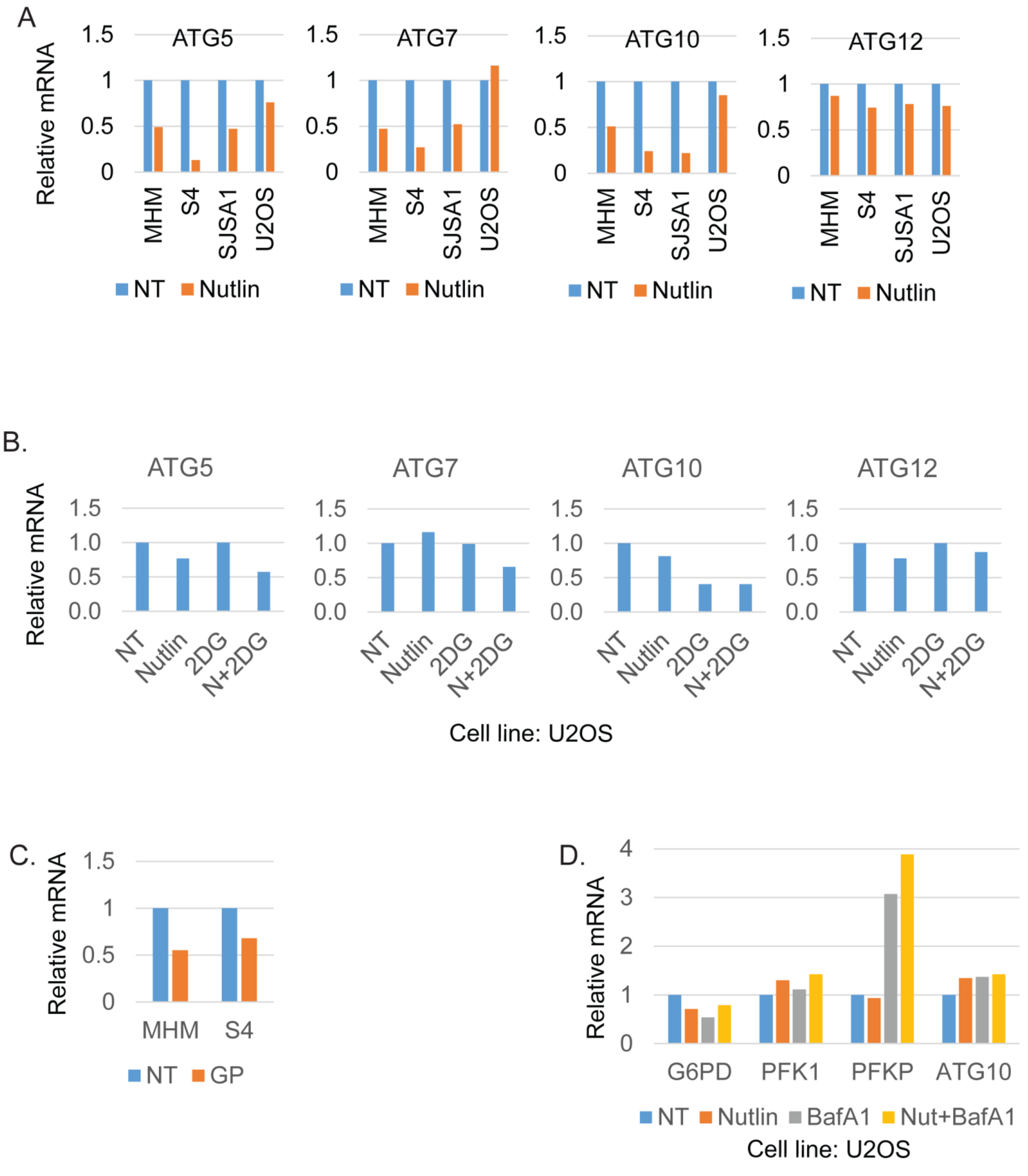

Supplemental Fig. 2. Nutlin downregulates ATG genes by inhibition of glycolysis in sensitive cells but not in resistant cells. A. The indicated cell lines were treated with vehicle (NT) or Nutlin for 24 hours and mRNA was quantitatively analyzed for the indicated gene expression. B. U2OS cells were treated with vehicle (NT) or Nutlin in the presence or absence of 2-DG (0.1 mM) for 24 hours and mRNA was quantitatively analyzed for the indicated gene expression. C. MHM and S4 cells were grown in 10 mM glucose (NT) or 0.1 mM glucose (GP) for 12 hours and mRNA was quantitatively analyzed for ATG10 gene expression. D. U2OS cells were treated with vehicle, Nutlin, Bafilomycin A1, or Nutlin plus Bafilomycin A1 for 24 hours and mRNA was quantitatively analyzed for the indicated gene expression.

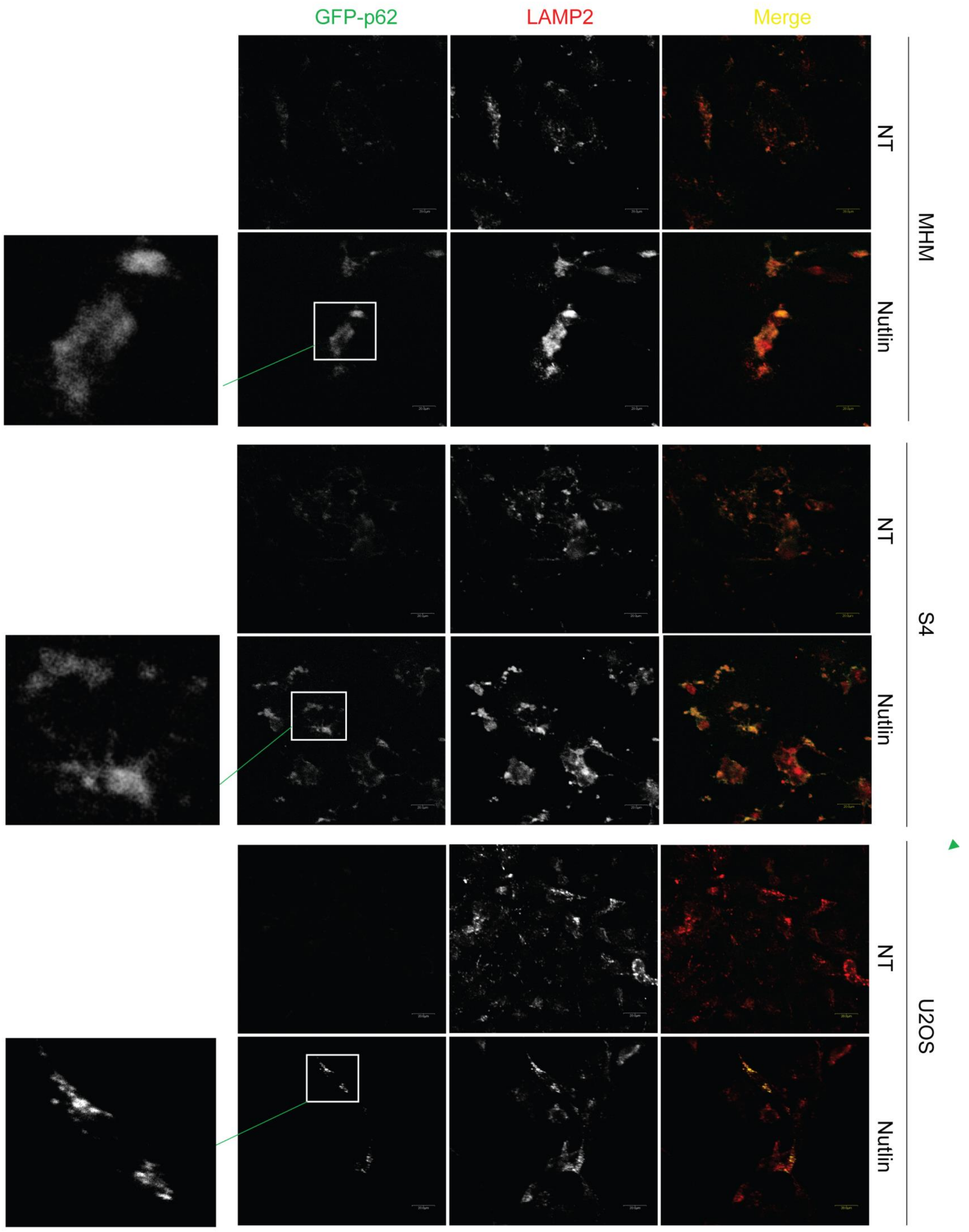

Supplemental Fig. 3. Nutlin induces formation of p62 aggregates in sensitive cells. A. MHM, S4, and U2OS cells were transfected with GFP-p62 and then treated with vehicle (NT) or nutlin for 24 hours. The cells were then immunostained for LAMP2 (red) and scanned with a confocal microscope. Note that in MHM and S4 cells Nultin treatment increased fluorescence intensity of GFP-p62 which form patches which are different from U2OS cells in which GFP-p62 show punctate staining which colocalize with LAMP2 labeled lysosomes.

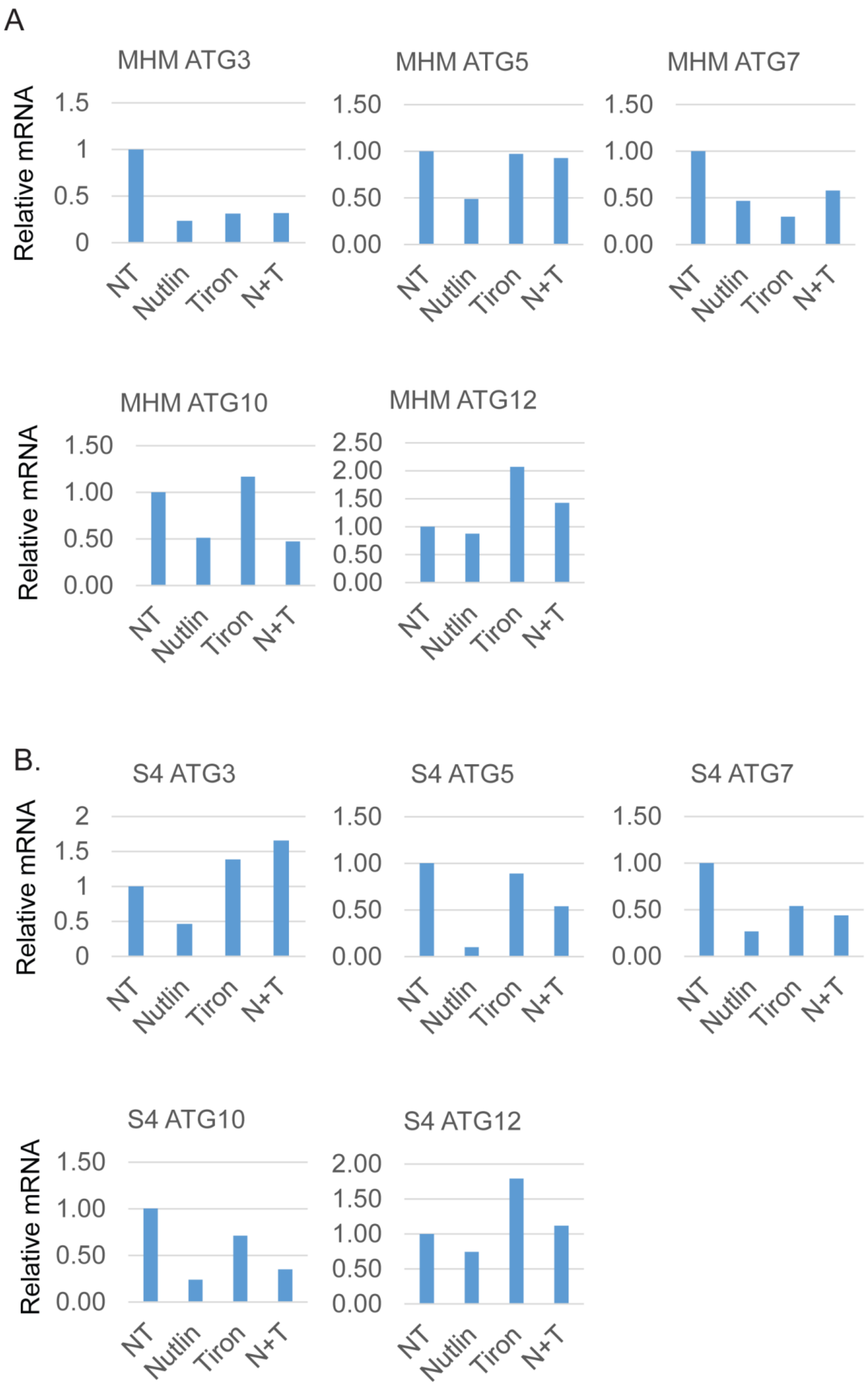

Supplemental Fig. 4. Superoxide scavenging maintains ATG genes. MHM (A) and S4 (B) cells were treated with vehicle (NT) or Nutlin for 24 hours. mRNA was quantitatively analyzed for the indicated gene expression.

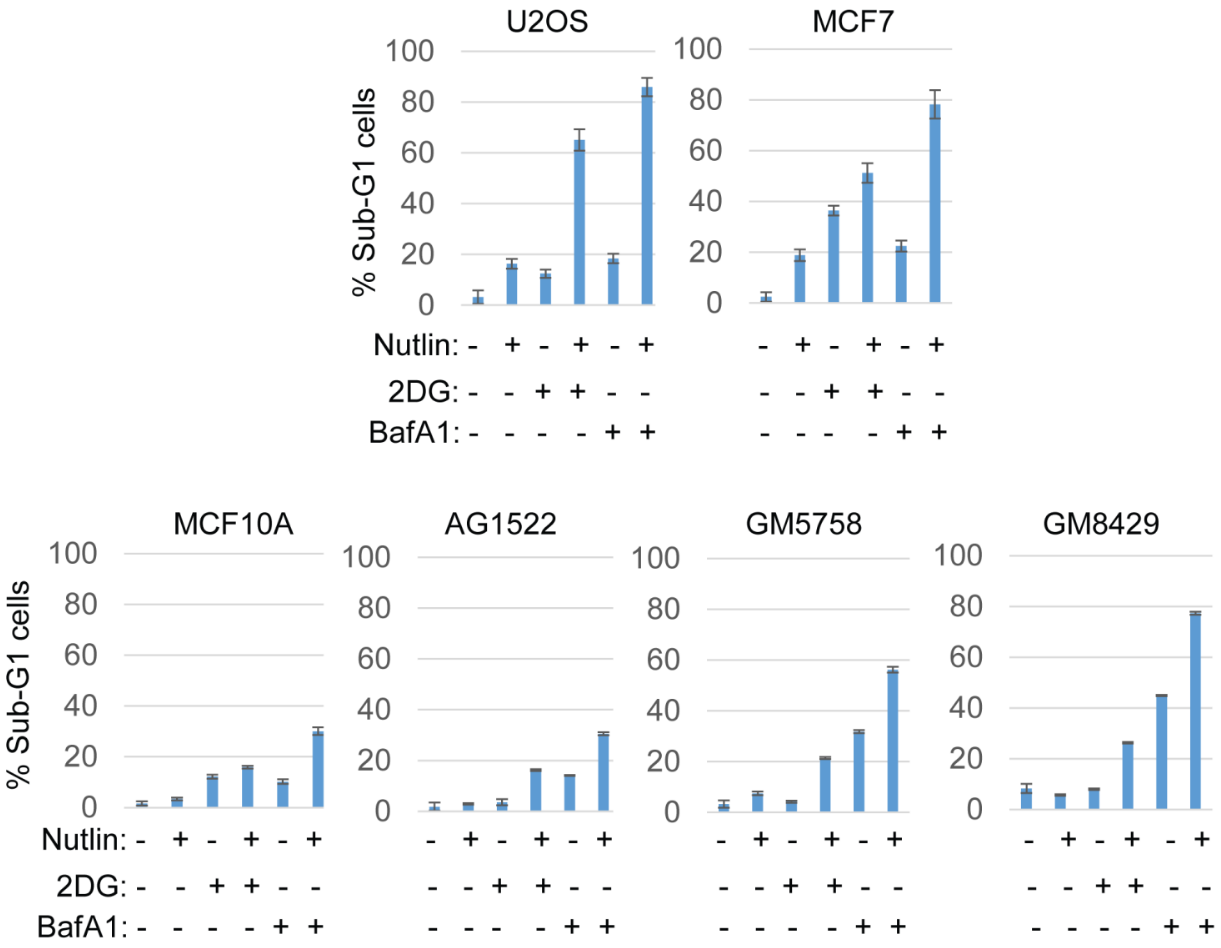

Supplemental Fig. 5. Non-cancerous cells were less sensitized by 2DG and Bifilomycin A1 to Nutlin-induced apoptosis. The indicate cell lines were treated with vehicle, Nultin, 2DG, Bifilomycin A1, and combination of Nutlin with 2DG or Bifilomycin A1 for 72 hours and analyzed for sub-G1 cells.

Supplemental Table 1-Primers for real-time PCR analysis

Gene	Forward	Reverse
ATG3	5'-ACATGGCAATGGGCTACAGG-3'	5'-CTGTTTGCACCGCTTATAGCA-3'
ATG5	5'-AAAGATGTGCTTCGAGATGTGT-3'	5'-CACTTTGTCAGTTACCAACGTCA-3'
ATG7	5'-ATGATCCCTGTAACTTAGCCCA-3'	5'-CACGGAAGCAAACAACTTCAAC-3'
ATG10	5'-AGACCATCAAAGGACTGTTCTGA-3'	5'-GGGTAGATGCTCCTAGATGTGAC-3'
ATG12	5'-CTGCTGGCGACACCAAGAAA-3'	5'-CGTGTTCGCTCTACTGCCC-3'
G6PD	5'CGAGGCCGTCACCAAGAAC-3'	5'-GTAGTGGTCGATGCGGTAGA-3'
HKII	5'-TTGACCAGGAGATTGACATGGG-3'	5'-CAACCGCATCAGGACCTCA-3'
PFK1	5'-ATTGCGGTTTTCGATGCCAC-3'	5'-GCCACAACTGTAGGGTCGT-3'
PGAM2	5'-AGAAGCACCCCTACTACAACTC-3'	5'-TCTGGGGAACAATCTCCTCGT-3'
β-Actin	5'-TCGTGCGTGACATTAAGGAG-3'	5'-GTCAGGCAGCTCGTAGCTCT-3'
